# Hospital-family collaborative DTT intervention to reduce the parenting stress through improving core symptoms and family functioning in children with autism spectrum disorder: a randomized controlled trial

**DOI:** 10.3389/fped.2025.1708217

**Published:** 2025-12-05

**Authors:** Mengqin Dai, Juan Li, Xiaohong Chen, Lian Chen, Xiaocui Tang, Haixia Yu, Yun Qiu, Yuwei Yang

**Affiliations:** Mianyang Central Hospital, School of Medicine, University of Electronic Science and Technology of China, Mianyang, China

**Keywords:** autism spectrum disorders, child, caregiver, gesell developmental schedules, parenting stress index, family assessment device (FAD)

## Abstract

**Introduction:**

Autism spectrum disorders (ASD) have emerged as a globally recognized public health concern. Currently, discrete trial teaching (DTT) is an effective intervention approach for ASD rehabilitation in hospitals. However, family-based interventions often yield limited outcomes. This study aims to develop a hospital-family collaborative DTT program guided by King's goal attainment theory, to support parents in delivering continuous and effective intervention within home environments.

**Method:**

This single-blind randomized controlled study included 84 children with ASD aged 1 to 6 years. Participants were stratified by gender and age and randomly assigned to either the experimental group (*n* = 42) or the control group (*n* = 42) using a random number table. The experimental group received a hospital-family collaborative DTT program, consisting of one month of hospital intervention followed by three months of family-based intervention, while the control group received standard DTT rehabilitation. Outcomes were assessed using the Gesell Developmental Schedules (GESELL), Parenting Stress Index-Short Form (PSI-SF), Family Assessment Device (FAD), along with DTT theoretical and skill evaluations.

**Results:**

Except that the PSI scores were unaffected by the intervention method, the GESELL, PSI, FAD, theoretical, and skill scores were significantly influenced by both intervention time (*F* = 37.70–896.12, all *P* < 0.001), intervention method (*F* = 37.70–896.12, all *P* < 0.001), and their interaction (*F* = 5.83–75.27, all *P* < 0.01). Partial correlation analysis revealed that improvements in parenting stress were initially linked to changes in “adaptive” items on the GESELL and FAD scales during the hospital intervention phase (Δ FAD. affective reaction: r_partial_ = 0.225, *P* = 0.043; Δ GESELL. adaptation behavior: r_partial_ = −0.290, *P* = 0.009; Δ parental knowledge: r_partial_ = −0.432, *P* < 0.001), followed by improvements in “behavioral” items during the family-based intervention phase (ΔFAD. problem-solving: r_partial_ = 0.433, *P* < 0.001; ΔGESELL. gross motor behavior: r_partial_ = −0.292, *P* = 0.010; *Δ*GESELL. fine motor behavior: r_partial_ = −0.309, *P* = 0.012; ΔGESELL. personal-social behavior: r_partial_ = −0.327, *P* = 0.001). For all participants, extremely high levels of parenting stress were independently associated with FAD disorders (particularly in problem-solving, affective responsiveness, and affective involvement), child factors (including male, language disorder, and attention-deficit/hyperactivity disorder), caregiver factors (including male, lower education level, and unversed DTT skills), as well as conventional DTT programs and shorter intervention durations (all *P* < 0.05).

**Discussion:**

Our hospital-family collaborative DTT program significantly improved children's ASD symptoms, family function, and parenting stress, demonstrating the value of ongoing family-based DTT intervention. The improvements in children's symptoms and family function showed a time-dependent shift from adaptive to behavioral changes, which were linked to lower parental stress.

## Introduction

Autism spectrum disorders (ASD) are a neurodevelopmental disorder primarily characterized by social communication impairments, repetitive stereotyped behaviors, and sensory perception abnormalities (1). In recent years, the prevalence of ASD has been steadily rising, and it has now become a widely recognized public health concern globally, placing a significant burden on both families and society. The latest data from the United States show that ASD incidence has increased from 1 in 45 to 1 in 36 (2, 3). In China, surveys indicate that about 0.7% of children have autism, making it the most common mental disability among children (4). There are currently no effective drugs for ASD; rehabilitation training remains the primary intervention (5), often requiring long-term or lifelong commitment and significant costs (6). As a result, parents not only have to confront the abnormal behaviors of children with ASD, but also have to bear multiple pressures and challenges on family life (7). Moreover, parents of ASD children experience higher stress levels compared to those with other disabilities like Down syndrome, cerebral palsy, or intellectual disabilities (8), which also negatively affect the rehabilitation interventions.

As the prevalence of ASD continues to rise, its rehabilitation strategies have become a top focus (9). Discrete trial teaching (DTT) is widely recognized as an effective intervention for children with ASD. This approach incorporates the core principles of applied behavior analysis (10) to break down training objectives into manageable steps and teach and strengthen the mastery of targeted skills through four simple and repetitive ways: instruction, response, outcome, and pause. Repeat these steps and adjust as needed until the trainees master the intended skills (11). Numerous studies have shown that DTT can significantly improve the social, self-care, and cognitive skills of children with ASD, reduce problem behaviors, and enhance social adaptability, thereby promoting overall development (12–14). However, most existing evidence focuses on short-term interventions conducted in clinical settings. There is limited empirical support for how to transform hospital-based DTT into continuous family-based interventions and integrate professional practices with daily life (15). Therefore, more research is needed to explore the family generalization and long-term maintenance of DTT intervention measures.

To achieve an effective implementation effect of family-based DTT interventions, the focus should be on how well family members master DTT measures. King's goal achievement theory, developed by American nursing theorist Imogene M. King, is a conceptual framework that emphasizes interpersonal interactions to achieve shared goals. As a patient-centered nursing theory, it can promote physical well-being through dynamic exchanges between nurses and patients. The theory encourages mutual goal-setting and active participation from both parties to achieve effective care outcomes (16). Due to its emphasis on prioritizing patients' perspectives, fostering interactive communication, and progressing incremental education, it has proven effective in improving self-management among patients with chronic conditions like diabetes, myocardial infarction, and chronic obstructive pulmonary disease (17–19). Yet, it has not been applied to ASD management or family caregiving education.

A meta-analysis and systematic review have confirmed that the current family-based interventions for ASD remain suboptimal, suggesting the need to incorporate professional skills into daily family interventions (20, 21). To more effectively reduce ASD symptoms, ease family financial and emotional burdens, and fill the gap in clinical evidence, this study integrates King's goal achievement theory with DTT teaching to support parents in delivering consistent, effective interventions in daily life.

## Method

### Ethical review

This study was a single-blind randomized controlled trial, and registered in the National Universal Health Security Platform (Registration No.: MR-51-25-036154, Date: May 12, 2025). The study followed the 2013 revised Declaration of Helsinki and was approved by the Medical Ethics Committee of Mianyang Central Hospital (Approval No.: S20250313-01, Date: Feb. 12, 2025). All parents of children with ASD signed informed consent.

### Participants

During March 2025 to July 2025, a total of 84 children with ASD and their primary caregivers were recruited from the Department of Developmental and Behavioral Pediatrics in Mianyang Central Hospital School of Medicine, University of Electronic Science and Technology of China. The inclusion criteria were as follows: (1) Child aged 1 to 6 years, accompanied by primary caregiver aged 20 to 65 years; (2) Child who were firstly diagnosed with ASD from a specialist physician, based on the diagnostic criteria outlined in the Diagnostic and Statistical Manual of Mental Disorders, Fifth Edition (22); (3) Primary caregiver who had no mental disorders and could communicate normally; (4) Parent and primary caregiver were willing to participate in the study and had provided written informed consent. The exclusion criteria were as follows: (1) Child or primary caregiver had other serious diseases, such as serious behavioral disorder, mutism, Tic disorder, and epilepsy; (2) Child and/or primary caregiver had recently received other forms of systematic rehabilitation training; (3) Child was unable to cooperate, or primary caregiver lacked sufficient language proficiency to understand DTT instructions; (4) child who receive any form of therapy other than DTT during the study period.

According to a pre-prepared random number table stratified by gender (male and female) and age (1–3 years and 4–6 years), children and their primary caregivers were assigned to either the control group or the experimental group, following the order of their visits. Based on the research design, the sample size was estimated using the repeated measures module in PASS software. With a significance level (α) set at 0.05 and statistical power (1−β) set at 90% (β = 0.10), and incorporating the Huynh-Feldt and Greenhouse-Geisser corrections to account for potential violations of sphericity, the minimum required sample size per group was determined to be 28 participants. To accommodate an anticipated dropout rate of 30%, the adjusted sample size per group was calculated to be at least 37 participants.

### Comparative implementation of conventional and collaborative DTT training

#### Establishment of two independent DTT intervention teams

Each comprised three members, including one developmental and behavioral pediatrician and two ASD specialist nurses. Each team received training on the DTT intervention, delivered either in the conventional mode or through the hospital-family program. The pediatrician was responsible for conducting the ASD diagnosis and initial evaluation, developing the DTT intervention plan with parents, and supervising the subsequent follow-up visits. Specialist nurses were responsible for conducting comprehensive evaluations of developmental level and disorder severity, assisting pediatricians to design personalized rehabilitation plans with primary caregivers, training primary caregivers in DTT intervention skills, and providing guidance for family-based DTT rehabilitation after discharge. Each team was allocated an independent diagnostic room and a dedicated rehabilitation training room to minimize cross-group interference. Appropriate teaching materials and assistive tools were provided within the dedicated rehabilitation room. Additionally, a shared supervision nurse, who remained blinded to the group allocation, was responsible for evaluating the DTT skill proficiency of primary caregivers and collecting scale-based survey data regarding the rehabilitation outcomes.

#### Conventional training program for the control group

Specialized nurses conducted a one-month hospital DTT intervention, and primary caregivers implemented three-month daily DTT exercises after in-class autonomous learning. During the hospital DTT intervention, a rehabilitation plan is jointly formulated by specialist nurses and a specialist doctor following a comprehensive assessment of the child's developmental level as well as the severity of social, language, and behavioral impairments. Subsequently, specialist nurses conducted one-on-one DTT sessions for children with ASD five days per week, with each session lasting 45 min. DTT training broke down targeted skills into small, manageable steps and delivered them through a structured instructional approach. Specific reinforcers, such as toys or snacks, were selected based on individual children's preferences to enhance their engagement and motivation during the learning process. Meanwhile, the specialist nurses provided in-class training and guidance to parents on DTT skills, covering how to break down training steps, formulate instructions, select and use reinforcers, and provide and adjust appropriate assistance. After the hospital intervention ended, primary caregivers continued the family-based DTT intervention in daily situations using the skills they learned in the hospital. Meanwhile, a nurse-caregiver WeChat group was created to support remote question-and-answer sessions. The family-based DTT intervention lasted three months, followed by a required outpatient follow-up visit.

#### King interactive achievement program for the experimental group

Based on the conventional DTT program for the control group, the King interactive attainment system was integrated to establish a hospital-family collaborative program. During the hospital DTT intervention, pediatricians, specialist nurses, and parents collaboratively developed a stepwise rehabilitation plan after thoroughly completing a comprehensive assessment of the child's condition and gathering relevant information (including parental personality, age, cultural background, family support, psychological characteristics, understanding of disease-related knowledge, and living environment). Subsequently, specialist nurses delivered DTT courses to children with ASD and their primary caregivers at the same frequency and duration.

During the first five days of hospital intervention, primary caregivers received 45 min training sessions each day, including two days of theory and three days of practice. Theoretical teaching encompassed key components such as task breakdown, instruction delivery, reinforcer selection and use, appropriate assistance provision, and determination of assistance levels. Practical training was conducted through one-on-one demonstrations by specialized nurses to enhance caregivers' proficiency in DTT skills. Meanwhile, a pediatrician-nurse-caregiver WeChat group was created to support remote question-and-answer sessions. Additionally, the pediatrician shared several rehabilitation training courses within the WeChat group to enhance caregivers' understanding of rehabilitation and to support the development of their intervention skills. These courses included topics such as “Children's Related Behaviors and Descriptions”, “Basic Knowledge of ASD and Analysis of Common Problem Behaviors”, and “Functional Games for Children with ASD”.

Near the end of the hospital intervention, the pediatrician, nurses, and caregivers formulated a family-based DTT rehabilitation plan together. During the home intervention, caregivers uploaded weekly videos to the WeChat group, showing the implementation of DTT in daily routines. Based on the rehabilitation condition, nurses adjusted the plan promptly and guided caregivers in conducting progressive rehabilitation training. Near the end of the hospital intervention, doctors, nurses, and parents jointly formulated a family DTT rehabilitation plan. During the family intervention period, the main caregiver uploaded videos of implementing DTT in daily situations to the WeChat group every week. The specialist nurse promptly adjusted the rehabilitation plan according to the child's rehabilitation progress and guided the caregivers to carry out progressive rehabilitation training. Additionally, caregivers were encouraged to share their experiences to enhance their confidence throughout the family-based rehabilitation process.

### Investigation and evaluation of intervention effects

The survey content included the Gesell Developmental Schedules (GESELL), Family Assessment Device (FAD), Parenting Stress Index Short Form (PSI-SF), as well as the ASD theoretical examination and DTT skill assessment. The first three scales were investigated before intervention, one month after hospital intervention, and three months after family-based intervention. The ASD theoretical examination and DTT skill assessment were conducted at one week into hospital intervention, one month after hospital intervention, and three months after family-based intervention. Except for the pre-intervention GESELL assessment conducted by a specialist nurse, all other assessments were performed by the shared supervision nurse who remained blinded to the group allocation. At the time of pre-intervention GESELL assessment, the specialist nurse recorded the sociodemographic characteristics of the children and their primary caregivers, including the caregivers' gender, age, kinship, educational level, occupation, monthly family income, as well as the children's gender, physical age, age at diagnosis, and accompanying symptoms.

The GESELL scale was used to evaluate children's developmental levels across five dimensions: adaptation behavior, gross motor behavior, fine motor behavior, language behavior, and personal-social behavior, encompassing a total of 97 items. For each dimension, the developmental quotient (DQ) was calculated based on the child's performance using the formula: developmental age/chronological age × 100. Developmental Age refers to the age level that corresponds to the child's observed developmental stage. A DQ score of each dimension above 85 was considered within the normal developmental range, 76–85 indicated a borderline state, 55–75 indicated mild developmental delay, 40–54 indicated moderate developmental delay, and <40 indicated severe developmental delay. The Cronbach's α coefficient of five dimensions was reported as 0.85 to 0.95 (23).

The FAD scale comprised 60 items distributed across seven dimensions: problem-solving (6 items), communication (9 items), role (11 items), affective responsiveness (6 items), affective involvement (7 items), behavior control (9 items), and general functioning (12 items). Each item was rated on a 4-Likert scale, ranging from 1 (completely agree) to 4 (completely disagree) for positive items, while 1 (completely disagree) to 4 (completely agree) for negative items. Higher scores indicated poorer family functioning. The Cronbach's α coefficients for the scale range from 0.78 to 0.86 (24).

The PSI-SF scale comprised three dimensions: parental distress, parenting difficulty, and difficult child, each consisting of 12 items. Each item was rated on a 5-Likert scale ranging from 1 (strongly disagree) to 5 (strongly agree), with higher total scores indicating higher levels of parenting stress. The level of parenting stress was classified into four categories: a total score of ≤85, 86–90, 91–98, and ≥99 indicated normal, borderline, high, and very high stress, respectively. This scale demonstrated strong internal consistency, with reliability coefficients exceeding 0.90, which reflects good reliability and validity (25).

Caregivers' mastery of DTT was evaluated through a self-made theoretical test (including single-choice, multiple-choice, and case analysis) and operational assessment, both scored out of 100.

### Statistical analysis

Data analysis was processed using SPSS v25.0 software (IBM Corp., Armonk, NY, USA) and MedCalc v22.021 software (MedCalc Software, Mariakerke, Belgium). Quantitative data were expressed as mean ± standard deviation. The inter-group differences at the same time point were analyzed using the independent sample *t*-test (if equal variance) or Welch *t*-test (if unequal variance). For comparisons of inter-group differences across multiple time points, repeated measures ANOVA was employed, and the adjusted *P*-value for the effect value was determined by the Huynh-Feldt correction (if epsilon > 0.75) or the Greenhouse-Geisser correction (if epsilon < 0.75). Count data were expressed as frequency (percentage), and the inter-differences were compared using the Chi-square test. The significance level was set at α = 0.05 for two-tailed hypothesis testing.

## Result

### The sociodemographic characteristics of the participants

The sociodemographic characteristics of 84 children with ASD and their caregivers are listed in Table 1. No statistically significant differences were observed in any of these characteristics between the experimental and control groups (all *P* > 0.05).

**Table 1:** The sociodemographic characteristics of children with ASD and their caregivers.

Item	Category	All [*n* (%)]	Experimental [*n* (%)]	Control [*n* (%)]	*χ* ^2^	*P*
Patient						
Gender	Male	40 (47.6)	15 (35.7)	20 (47.6)	1.210	0.271
	Female	44 (52.4)	27 (64.3)	22 (52.4)		
Physical age	1–3 years	30 (35.7)	12 (28.6)	18 (42.8)	1.844	0.174
	4–6 years	54 (64.3)	30 (71.4)	24 (57.2)		
Age at diagnosis	1–3 years	37 (44.0)	19 (45.2)	18 (42.8)	0.048	0.827
	4–6 years	47 (56.0)	23 (54.8)	24 (57.2)		
Comorbidities						
Mental retardation	Yes	59 (70.2)	30 (71.4)	29 (69.1)	0.056	0.812
	No	25 (29.8)	12 (28.6)	13 (30.9)		
ADHD	Yes	51 (60.7)	24 (57.1)	27 (64.3)	0.444	0.505
	No	33 (39.3)	18 (42.9)	15 (35.7)		
Epilepsy	Yes	68 (80.9)	33 (78.6)	35 (83.3)	0.305	0.581
	No	16 (19.1)	9 (21.4)	7 (16.7)		
Language disorder	Yes	23 (27.4)	12 (28.6)	11 (26.2)	0.059	0.808
	No	61 (72.6)	30 (71.4)	31 (73.8)		
SID	Yes	56 (66.7)	28 (66.7)	28 (66.7)	0.000	1.000
	No	28 (33.3)	14 (33.3)	14 (33.3)		
Primary caregiver						
Gender	Male	24 (28.6)	14 (33.3)	10 (23.8)	0.922	0.367
	Female	60 (71.4)	28 (66.7)	32 (76.2)		
Age	20–30 years	18 (21.4)	11 (26.2)	7 (16.7)	1.356	0.716
	31–40 years	45 (53.6)	22 (52.4)	23 (54.7)		
	41–50 years	12 (14.3)	5 (11.9)	7 (16.7)		
	≥51 years	9 (10.7)	4 (9.5)	5 (11.9)		
Kinship	Father	20 (23.8)	12 (28.6)	8 (19.1)	1.075	0.584
	Mother	55 (65.5)	26 (61.9)	29 (69.0)		
	Grandparent	9 (10.7)	4 (9.5)	5 (11.9)		
Education	High school	39 (46.4)	17 (40.5)	22 (52.4)	1.182	0.277
	College or above	45 (53.6)	25 (59.5)	20 (47.6)		
Occupation	White-collar worker	36 (42.9)	18 (42.9)	15 (35.7)	0.928	0.629
	Manual worker	23 (27.4)	11 (26.2)	15 (35.7)		
	Retired/unemployed	25 (29.7)	13 (30.9)	12 (28.6)		
Monthly family income	<10,000 RMB	53 (63.1)	29 (69.1)	24 (57.1)	1.363	0.506
	10,000–20,000 RMB	20 (23.8)	8 (19.0)	12 (28.6)		
	>20,000 RMB	11 (13.1)	5 (11.9)	6 (14.3)		

ADHD, attention-deficit/hyperactivity disorder; SID, sensory integration dysfunction.

### Comparison of GESELL, PSI-SF, and FAD scores between the two intervention groups

The inter-group difference analysis at the same time point showed (Table 2): Before the intervention, except for the AR dimension scores, no statistically significant differences were observed between the two groups in the total scores and dimension scores of GESELL, PSI-SF, and FAD scales (all *P* > 0.05). At one month after hospital intervention, statistically significant differences emerged between the two groups in the total scores and GMB, FMB, and PSB dimension scores of GESELL scale (*t* = −3.33 to −4.42, all *P* < 0.01), as well as in the total scores and CM, RL, AR, AI, and BC dimension scores of FAD scale (*t* = 2.81 to 4.24, all *P* < 0.01). At three months after family-based intervention, except for the LB dimension scores, statistically significant differences were observed between the two groups in the total scores and dimension scores of GESELL, PSI-SF, and FAD scales (all *P* < 0.05).

**Table 2:** The GESELL, PSI-SF and FAD scores before intervention, during in-hospital intervention (the first month) and during home intervention (the third month).

Scale	Pre-intervention			Hospital intervention			Family intervention			Repetitive measurement ANOVA**		
	Con (*n* = 42)	Exp (*n* = 42)	*t*-test*	Con (*n* = 42)	Exp (*n* = 42)	*t*-test*	Con (*n* = 42)	Exp (*n* = 42)	*t*-test*	Intervention method	Intervention duration	Interaction
GESELL	325.7 ± 10.5	323.1 ± 10.7	1.16, 0.248	331.0 ± 11.8	345.2 ± 16.0	−4.42, < 0.001	344.5 ± 16.0	364.9 ± 17.2	−5.61, <0.001	16.67, <0.001	245.12, <0.001	37.69, <0.001
AB	68.5 ± 2.3	67.9 ± 2.4	1.12, 0.268	69.4 ± 4.4	71.1 ± 5.4	−1.23, 0.222	72.5 ± 5.9	76.6 ± 7.3	−2.84, 0.006	5.71, 0.019	46.37, <0.001	5.83, 0.008
GMB	72.0 ± 1.8	71.5 ± 1.7	1.23, 0.224	73.0 ± 3.9	76.6 ± 5.5	−3.34, 0.001	76.1 ± 7.1	81.2 ± 7.3	−3.23, 0.002	14.48, 0.001	43.48, <0.001	7.86, 0.002
FMB	65.0 ± 2.0	64.2 ± 2.1	1.69, 0.095	65.0 ± 4.4	69.0 ± 5.8	−3.33, 0.001	68.6 ± 6.4	72.6 ± 5.8	−3.02, 0.003	13.07, 0.001	38.63, <0.001	8.27, 0.001
LB	57.8 ± 2.4	57.2 ± 2.6	1.29, 0.199	59.8 ± 4.6	60.5 ± 4.7	−0.51, 0.612	61.8 ± 5.2	63.5 ± 6.2	−1.33, 0.188	0.69, 0.408	37.70, <0.001	1.95, 0.157
PSB	62.8 ± 2.3	62.5 ± 2.3	0.48, 0.634	63.8 ± 4.0	68.0 ± 4.6	−4.30, <0.001	65.6 ± 5.4	71.1 ± 8.1	−3.67, <0.001	31.96, 0.001	110.00, <0.001	0.29, 0.709
PSI-SF	116.4 ± 21.9	121.6 ± 22.3	−1.09, 0.278	105.9 ± 20.9	100.0 ± 18.6	1.38, 0.170	92.7 ± 19.4	78.8 ± 15.2	3.66, <0.001	1.33, 0.253	896.12, <0.001	75.27, <0.001
PD	39.1 ± 8.1	41.1 ± 8.3	−1.09, 0.279	35.5 ± 8.8	33.6 ± 7.4	1.05, 0.297	30.9 ± 8.5	27.0 ± 5.7	2.46, 0.016	0.58, 0.449	421.43, <0.001	29.59, <0.001
DI	38.3 ± 8.9	39.9 ± 9.3	−0.84, 0.404	34.5 ± 8.4	32.9 ± 7.8	0.91, 0.364	30.1 ± 7.3	25.5 ± 6.0	3.17, 0.002	0.82, 0.368	385.12, <0.001	30.16, <0.001
DC	39.0 ± 7.8	40.6 ± 8.3	−0.94, 0.352	36.0 ± 8.1	33.4 ± 6.9	1.57, 0.121	31.9 ± 7.8	26.5 ± 6.1	3.49, 0.001	1.77, 0.187	308.63, <0.001	33.69, <0.001
FAD	139.7 ± 18.9	135.7 ± 19.9	0.95, 0.345	127.2 ± 17.8	111.9 ± 16.5	4.09, <0.001	110.8 ± 15.6	97.3 ± 14.4	4.11, <0.001	8.72, 0.004	530.71, <0.001	59.54, <0.001
PS	13.4 ± 2.9	14.1 ± 3.0	−1.14, 0.259	12.3 ± 2.8	11.8 ± 2.6	0.81, 0.422	10.9 ± 2.5	9.4 ± 2.6	2.64, 0.010	0.48, 0.490	416.37, <0.001	39.82, <0.001
CM	20.5 ± 4.4	18.9 ± 4.5	1.61, 0.110	18.7 ± 4.6	15.5 ± 3.7	3.44, 0.001	16.5 ± 4.2	13.6 ± 3.5	3.45, 0.001	8.26, 0.005	286.81, <0.001	9.25, 0.001
RL	25.0 ± 4.9	23.5 ± 5.2	1.33, 0.186	22.7 ± 4.6	19.4 ± 4.4	3.47, 0.001	19.4 ± 4.3	16.6 ± 4.1	3.03, 0.003	7.05, 0.010	302.91, <0.001	7.24, 0.002
AR	14.7 ± 2.6	13.5 ± 2.3	2.14, 0.035	13.4 ± 2.7	11.2 ± 1.9	4.24, <0.001	11.7 ± 2.6	9.6 ± 1.9	4.27, <0.001	13.97, <0.001	268.47, <0.001	7.69, 0.002
AI	17.0 ± 3.4	15.9 ± 3.5	1.50, 0.138	15.7 ± 3.6	13.2 ± 3.1	3.43, 0.001	13.7 ± 3.5	11.3 ± 2.8	3.45, 0.001	8.10, 0.006	376.89, <0.001	13.88, <0.001
BC	21.4 ± 3.5	20.6 ± 4.1	1.04, 0.301	19.1 ± 4.0	16.9 ± 3.3	2.81, 0.006	16.6 ± 3.7	14.7 ± 2.9	2.63, 0.010	5.02, 0.028	323.99, <0.001	6.04, 0.006
GF	27.7 ± 4.4	29.1 ± 5.0	−1.37, 0.175	25.5 ± 5.1	23.9 ± 4.3	1.57, 0.121	22.1 ± 4.7	20.0 ± 3.9	2.23, 0.029	0.65, 0.422	363.42, <0.001	24.13, <0.001

* Comparison between the two intervention groups at the same time point, the statistics are t and P.

** Effect analysis of repeated measures ANOVA, the statistics are F and P. The intervention duration influenced all observed indicators, while the intervention method and its interaction with duration affected most of the observed indicators. GESELL, gesell developmental schedules; AB, adaptation behavior; GMB, gross motor behavior; FMB, fine motor behavior; LB, language behavior; PSB, personal-social behavior; PSI-SF, parenting stress index short form; PD, parental distress; DI, parenting difficulty; DC, difficult child; FAD, family assessment device; PS, problem-solving; CM, communication; RL, role; AR, affective responsiveness; AI, affective involvement; BC, behavior control; GF, general functioning.

The repeated measures ANOVA analysis revealed (Table 2): During the hospital-family collaborative intervention, the total scores and AB, GMB, FMB, and PSB dimension scores of GESELL scale were affected by the intervention method (*F* = 5.71 to 31.96, all *P* < 0.05), as well as the total scores and CM, RL, AR, AI, and BC dimension scores of FAD scale (*F* = 5.02–13.97, all *P* < 0.05); the total scores and each dimension scores of GESELL, PSI-SF, and FAD were all affected by the intervention duration (*F* = 37.70 to 896.12, all *P* < 0.001); except for the LB and PSB dimension scores, the total scores and each dimension scores of GESELL, PSI-SF, and FAD were also affected by the interaction of intervention method and duration (*F* = 5.83 to 75.27, all *P* < 0.01).

### Comparison of theoretical and operational scores between the two intervention groups

**Figure 1:**
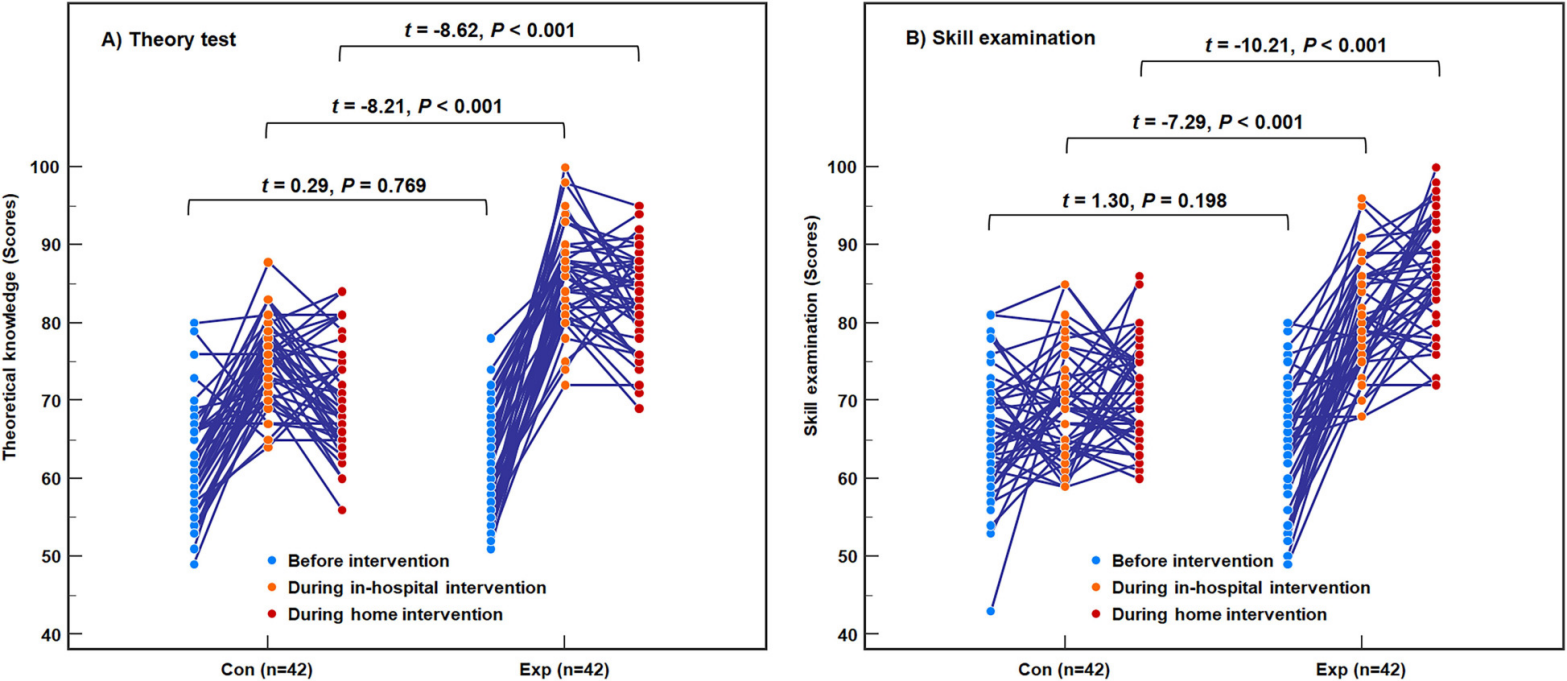
The theoretical and operational scores of the two intervention groups. **(A)** Theory test; **(B)** Skill examination. Con, the control group; Exp, the experimental group. There were no inter-group differences in theoretical and operational scores before the intervention, but differences emerged after the hospital intervention and persisted following three months of family-based intervention. The intervention method, duration, and their interaction all impacted on theoretical and operational scores.

The inter-group difference analysis at the same time point showed (Figure 1): Before the intervention, there were no statistically significant differences in the theoretical or operational scores between the two groups (*t* = 0.29 and 1.30, *P* = 0.769 and 0.198). Following one month of hospital intervention and three months of family-based intervention, both the theoretical and operational scores of the control group were significantly lower than those of the experimental group (*t* = −7.29 to −10.21, all *P* < 0.001). The repeated measures ANOVA analysis revealed that the intervention method (*F* = 53.56 and 52.92), duration (*F* = 269.88 and 96.59), and interaction between intervention method and duration (*F* = 33.91 and 40.68) all had significant impacts on theoretical and operational scores.

### Relationship between PSI-SF improvement and other evaluation improvements during hospital intervention and family-based intervention

We calculated the changes in scores for each scale and test in both the hospital and family-based interventions. For the hospital intervention, the score change was calculated as the difference between the hospital score and the pre-intervention score. For the family-based intervention, the score change was calculated as the difference between the family-based score and the hospital score. Using the PSI-SF score change as the dependent variable, and the score changes in each GESELL dimension, each FAD dimension, theory, and skill as covariates, the enter-method linear regression and partial correlation analysis revealed (Table 3): During hospital intervention, the ΔPSI-SF scores were positively correlated with those of ΔFAD.AR (affective reaction) (r_partial_ = 0.225, *P* = 0.043), while negatively correlated with those of ΔGESELL1.AB (adaptation behavior) and ΔTheory test (r_partial_ = −0.290 and −0.432, *P* = 0.009 and <0.001). During family intervention, the ΔPSI-SF scores were positively correlated with those of ΔFAD.PS (problem-solving) (r_partial_ = 0.433, *P* < 0.001), while negatively correlated with those of ΔGESELL gross motor behavior, ΔGESELL fine motor behavior, and ΔGESELL personal-social behavior (r_partial_ = −0.292, −0.309, and −0.327, *P* = 0.010, 0.012, and 0.001). All significances were confirmed by the stepwise method analysis. These findings suggest that moving from hospital to family-based interventions, the focus for children with ASD shifted from adaptive skills to behavioral outcomes, which significantly helped reduce parenting stress.

**Table 3:** Changes in PSI-SF scores in relation to changes in other evaluations during hospital and family-based interventions.

Intervention duration	Beta	SE	*r*_partial_	*t*	*P*	*VIF*
Hospital intervention						
ΔFAD.PS	0.743	0.781	0.114	0.951	0.345	1.633
ΔFAD.CM	0.668	0.533	0.149	1.252	0.215	1.650
ΔFAD.RL	−0.104	0.387	−0.032	−0.268	0.789	1.397
ΔFAD.AR	**1**.**111**	**0**.**541**	**0**.**225**	**2**.**054**	**0**.**043**	**1**.**197**
ΔFAD.AI	0.090	0.694	0.016	0.130	0.897	1.548
ΔFAD.BC	0.149	0.467	0.038	0.318	0.751	1.495
ΔFAD.GF	0.107	0.318	0.041	0.338	0.736	1.477
ΔGESELL.AB	**−0**.**500**	**0**.**186**	**−0**.**290**	**−2**.**691**	**0**.**009**	**1**.**437**
ΔGESELL.GMB	−0.237	0.165	−0.171	−1.439	0.155	1.175
ΔGESELL.FMB	−0.163	0.171	−0.114	−0.949	0.346	1.254
ΔGESELL.LB	−0.116	0.208	−0.067	−0.555	0.581	1.132
ΔGESELL.PSB	0.077	0.199	0.046	0.386	0.700	1.282
ΔTheory test	**−0**.**325**	**0**.**076**	**−0**.**432**	**−4**.**251**	**<0**.**001**	**1**.**420**
ΔSkill examination	−0.126	0.064	−0.215	−1.958	0.054	1.477
Family-based intervention						
ΔFAD.PS	**2**.**571**	**0**.**686**	**0**.**433**	**3**.**747**	**<0**.**001**	**1**.**264**
ΔFAD.CM	−0.572	0.564	−0.121	−1.014	0.314	1.476
ΔFAD.RL	−0.238	0.350	−0.081	−0.678	0.500	1.141
ΔFAD.AR	1.024	0.828	0.147	1.236	0.221	1.476
ΔFAD.AI	−0.346	0.612	−0.068	−0.565	0.574	1.277
ΔFAD.BC	−0.612	0.516	−0.141	−1.187	0.239	1.403
ΔFAD.GF	0.146	0.378	0.046	0.386	0.701	1.287
ΔGESELL.AB	−0.005	0.093	−0.007	−0.054	0.957	1.104
ΔGESELL.GMB	**−0**.**233**	**0**.**088**	**−0**.**292**	**−2**.**662**	**0**.**010**	**1**.**252**
ΔGESELL.FMB	**−0**.**259**	**0**.**101**	**−0**.**309**	**−2**.**572**	**0**.**012**	**1**.**401**
ΔGESELL.LB	−0.153	0.105	−0.174	−1.466	0.147	1.096
ΔGESELL.PSB	**−0**.**342**	**0**.**101**	**−0**.**327**	**−3**.**391**	**0**.**001**	**1**.**096**
ΔTheory test	0.024	0.098	0.030	0.246	0.806	1.256
ΔSkill examination	−0.038	0.080	−0.057	−0.474	0.637	1.170

From hospital interventions to family-based interventions, the closely associated factors contributing to the reduction of parental stress demonstrate a shift from adaptive skills to behavioral outcomes. Significance values are shown in bold. SE, standard error; VIF, variance inflation factor; FAD.PS, FAD.CM, FAD.RL, FAD.AR, FAD.AI, FAD.BC, and FAD.GF respectively represent the problem-solving, communication, role, affective responsiveness, affective involvement, behavior control, and general functioning dimensions of the Family Assessment Device; GESELL.AB, GESELL.GMB, GESELL.FMB, GESELL.LB, and GESELL.PSB respectively represent the adaptation behavior, gross motor behavior, fine motor behavior, language behavior, and personal-social behavior dimensions of the GESELL scale.

### Independent influencing factors of parenting stress degree

Finally, we summarized the three survey data. Using the PSI degree as the dependent variable, and incorporating other evaluation outcomes, the sociodemographic characteristics of children and their caregivers, along with the intervention method and duration as covariates, the stepwise multiple logistic regression analysis was conducted (Table 4). Compared with normal degree PSI, male patient with language disorder or attention-deficit/hyperactivity disorder, male caregiver and his/her lower education, conventional DTT, and short intervention duration (pre-intervention and hospital intervention) were risk factors for very high degree PSI group (OR = 3.586 to 41.791, all *P* < 0.05), while higher scores in DTT skill, FAD.PS (problem-solving), FAD.AR (affective responsiveness) and FAD.AI (affective involvement) were protective factors for the very high degree PSI group (OR = 0.534 to 0.892, all *P* < 0.05). For the high degree PSI group, the effects of all aforementioned factors were attenuated, as indicated by no significant effects of DTT skill, FAD4.AR, and language disorder (all *P* > 0.05), as well as the diminished risk effects (OR = 4.112 to 27.450, all *P* < 0.05) or protective effects (OR = 0.688 and 0.705, both *P* < 0.05) of the remaining factors. No significant influencing factors were found in the borderline degree PSI group (all *P* > 0.05).

**Table 4:** Independent factors related to the severity of parenting stress.

Influencing factor	Beta	SE	OR (95% CI)	Wald *χ*^2^	*P*
Borderline degree PSI					
Male patient	0.307	0.647	1.360 (0.383, 4.833)	0.226	0.635
Language disorder	0.767	0.707	2.152 (0.539, 8.602)	1.176	0.278
ADHD	−0.755	0.721	0.470 (0.114, 1.931)	1.097	0.295
Male caregiver	0.312	0.701	1.366 (0.345, 5.401)	0.198	0.657
High school or less	0.369	0.830	1.446 (0.284, 7.362)	0.198	0.657
DTT skill	−0.044	0.032	0.957 (0.899, 1.019)	1.872	0.171
FAD.PS	−0.133	0.114	0.875 (0.700, 1.094)	1.367	0.242
FAD.AR	−0.124	0.127	0.884 (0.689, 1.133)	0.952	0.329
FAD.AI	−0.114	0.111	0.892 (0.719, 1.108)	1.060	0.303
Conventional DTT	0.838	0.713	2.311 (0.571, 9.344)	1.381	0.240
Pre-intervention	2.544	1.363	12.725 (0.881, 183.895)	3.484	0.062
Hospital intervention	1.215	0.715	3.371 (0.830, 13.694)	2.887	0.089
Family-based intervention	Ref.	Ref.	Ref.	Ref.	Ref.
High degree PSI					
Male patient	**1** **.** **706**	**0**.**576**	**5.505** (**1.779, 17.039)**	**8**.**756**	**0**.**003**
Language disorder	0.032	0.705	1.033 (0.260, 4.110)	0.002	0.963
ADHD	**1**.**549**	**0**.**648**	**4.706** (**1.320, 16.774)**	**5**.**704**	**0**.**017**
Male caregiver	**1**.**555**	**0**.**675**	**4.735** (**1.262, 17.775)**	**5**.**309**	**0**.**021**
High school or less	**1**.**414**	**0**.**655**	**4.112** (**1.138, 14.860)**	**4**.**655**	**0**.**031**
DTT skill	−0.028	0.028	0.972 (0.921, 1.026)	1.034	0.309
FAD.PS	**−0**.**350**	**0**.**118**	**0.705** (**0.559, 0.889)**	**8**.**755**	**0**.**003**
FAD.AR	−0.121	0.131	0.886 (0.685, 1.146)	0.855	0.355
FAD.AI	**−0**.**374**	**0**.**106**	**0.688** (**0.559, 0.846)**	**12**.**474**	**<0**.**001**
Conventional DTT	**2**.**022**	**0**.**672**	**7.557** (**2.024, 28.217)**	**9**.**053**	**0**.**003**
Pre-intervention	**5**.**615**	**1**.**298**	**27.450** (**2.158, 349.233)**	**18**.**722**	**<0**.**001**
Hospital intervention	**2**.**941**	**0**.**717**	**18.943** (**4.645, 77.247)**	**16**.**822**	**<0**.**001**
Family-based intervention	Ref.	Ref.	Ref.	Ref.	Ref.
Very high degree PSI					
Male patient	**2**.**399**	**0**.**568**	**11.009** (**3.616, 33.516)**	**17**.**832**	**<0**.**001**
Language disorder	**1**.**277**	**0**.**610**	**3.586** (**1.084, 11.866)**	**4**.**377**	**0**.**036**
ADHD	**1**.**501**	**0**.**614**	**4.487** (**1.346, 14.952)**	**5**.**973**	**0**.**015**
Male caregiver	**2**.**537**	**0**.**655**	**12.645** (**3.504, 45.636)**	**15**.**013**	**<0**.**001**
High school or less	**2**.**306**	**0**.**653**	**10.034** (**2.792, 36.072)**	**12**.**480**	**<0**.**001**
DTT skill	**−0**.**283**	**0**.**125**	**0.754** (**0.590, 0.963)**	**5**.**134**	**0**.**023**
FAD.PS	**−0**.**628**	**0**.**116**	**0.534** (**0.425, 0.671)**	**29**.**129**	**<0**.**001**
FAD.AR	**−0**.**115**	**0**.**027**	**0.892** (**0.846, 0.940)**	**17**.**903**	**<0**.**001**
FAD.AI	**−0**.**521**	**0**.**103**	**0.594** (**0.486, 0.727)**	**25**.**573**	**<0**.**001**
Conventional DTT	**3**.**082**	**0**.**663**	**21.804** (**5.940, 80.039)**	**21**.**578**	**<0**.**001**
Pre-intervention	**8**.**338**	**1**.**301**	**41.791** (**3.260, 535.693)**	**41**.**041**	**<0**.**001**
Hospital intervention	**3**.**818**	**0**.**719**	**27.298** (**6.668, 111.749)**	**28**.**183**	**<0**.**001**
Family-based intervention	Ref.	Ref.	Ref.	Ref.	Ref.

Taking the normal degree PSI group as a reference, more influencing factors and their stronger effects were observed in the very high degree PSI group compared to those in the high degree PSI group. Significance values are shown in bold. The excluded covariates by stepwise analysis included parental theory score, five child factors (physical age, age at diagnosis, mental retardation, epilepsy, and sensory integration dysfunction), four caregiver factors (age, kinship, occupation, and monthly family income), four FAD dimensions (communication, role, behavior control, and general functioning dimensions), and all five GESELL dimensions. SE, standard error; OR, odds ratio; CI, confidence interval; PSI, parenting stress index; DTT, discrete trial teaching; ADHD, attention-deficit/hyperactivity disorder; FAD.PS, FAD.AR, and FAD.AI respectively represent the problem-solving, affective responsiveness, and affective involvement dimensions of the Family Assessment Device.

## Discussion

Although DTT intervention has been shown to significantly improve symptoms in children with ASD, family-based continuation interventions remain less effective (20, 21). This study aimed to develop a hospital-family collaborative DTT program based on King's goal attainment theory, to enhance caregivers' ability to continue interventions and assess its impact on ASD symptoms and family functioning. Our findings revealed a significant improvement in caregivers' skills through the hospital-family collaborative DTT program, which contributed to sustained benefits in children's ASD symptoms, family functioning, and a reduction in parenting stress. Specifically, during the hospital intervention phase, the program significantly extended caregivers' theoretical knowledge of ASD and improved parental affective responsiveness and children's adaptation behavior to reduce parenting stress; subsequently, in the family-based phase, improvements in parental problem-solving skills and children's gross motor behavior, fine motor behavior, and personal-social behavior further reduced parenting stress. The two-phase intervention illustrated a transition from “adaptive” to “behavioral” improvements in children's symptoms and family functioning, highlighting the critical role of family-based DTT intervention in the treatment process for children with ASD. Further influencing factor analysis revealed that parenting stress was impacted by child-related factors (including gender, language disorder, and ADHD), caregiver-related factors (including gender, education, and DTT skill), family functioning (including problem-solving, affective responsiveness, and affective involvement abilities), intervention approach, and intervention duration.

Our hospital-family collaborative DTT program is developed based on the King's goal attainment theory. This theory includes three levels: individual, interpersonal, and social systems. During the healthcare process, greater emphasis is placed on patients' active involvement in health management and their interaction with nurses, fostering mutual influence and collaborative efforts to achieve rehabilitation goals (26). At the individual system level, DTT, an intervention grounded in behavior analysis, can effectively enhance perceptual-motor integration in children with ASD through structured and repetitive training. This approach facilitates the establishment of stable learning patterns and improvement in core symptoms (12). At the interpersonal system level, parents who master core skills through interactive attainment learning can implement DTT training more accurately, improving parent-child interactions and clarifying family roles and communication. The active participation of family members can enhance overall family functioning, ensure the success of family-based interventions, and thus sustain the effects of the DTT intervention. At the social system level, ongoing guidance and support from medical professionals can provide families with essential resources. Professional involvement goes beyond traditional family-based interventions by offering parents reliable technical help and emotional support. Ultimately, this hospital-family collaboration successfully eases parental stress and anxiety during interventions.

Our program can effectively reduce parenting stress by improving ASD core symptoms in children and enhancing family functioning. Evidence supporting this conclusion includes: (1) consistently lower GESELL, FAD, and PSI-SF scores in the experimental group compared to the control group during intervention; (2) significant independent influence of increased caregivers' DTT skills on reducing parental stress levels; and (3) notable antagonistic effect of hospital-family collaboration program on both high and extremely high levels of parental stress. Overall, these findings support the effectiveness of the intervention program. Noteworthily, the intervention effect showed a clear time-dependent trend. With the successive implementation of hospital and family-based interventions, parental stress gradually decreased. Repeated measures ANOVA also revealed a significant effect of intervention time on the GESELL, PSI-SF, and FAD scores, as well as a significant interaction effect between method and time on most dimensions except for the LB and PSB dimensions. These findings further suggest that the hospital-family collaboration DTT program has advantages in treating children with ASD, and that integrating professional guidance with family-based intervention improves parental caregiving capabilities.

As the hospital and family-based interventions were successively implemented, the crucial factor influencing parenting stress shifted from “adaptive” to “behavioral” improvements in the child's symptoms and family functioning. During the hospital intervention phase, increased parenting stress was positively linked to the decline in affective responsiveness within the family. Affective responsiveness refers to the emotional reactions of family members to external stimuli (27), whose deterioration may be linked to parents' first learning how to identify and respond to their autistic children's emotional signals. Parents not only require greater cognitive and emotional engagement to acquire new skills, but they also often experience concerns about whether their emotional responses meet professional expectations when participating in the structured interventions, commonly referred to as evaluation anxiety. These findings highlight the importance of including psychological support for parents during the hospital intervention phase to reduce negative emotions linked to professional skill acquisition. Consistent with previous studies (28), improvements in children's adaptation behaviors and parents' knowledge of ASD were negatively correlated with increased parenting stress. Improving children's adaptive behaviors can reduce parental care burdens, alleviate objective stressors, and potentially mitigate the cascading effects of problem behaviors between parents and children. Meanwhile, parents' understanding of ASD intervention theories helps them better grasp their children's problem behaviors and avoid blaming themselves for parenting failures. Overall, during the hospital intervention phase, improvements in both children and their families reflected their ability to actively adapt to ASD treatment, which was closely linked to reduced parenting pressure.

Unlike the hospital intervention phase, the factors influencing the reduction in parental stress shifted from “adaptive” to “behavioral” improvements during the family-based intervention phase. Improvements in children's behavioral indicators (gross motor behavior, fine motor behavior, and personal-social behavior) were negatively correlated with increased parenting stress. As gross and fine motor behaviors develop, children become more independent, reducing the need for daily parental assistance. Development in personal-social behavior can enhance parent-child communication and ease frustration from social difficulties, effectively lowering parenting stress. Overall, better motor and social skills reduced children's problem behaviors, increased parental confidence, and strengthened rehabilitation motivation. Meanwhile, the deterioration of problem-solving ability in family functioning was also found to be linked to the increase in parenting stress. Problem-solving ability refers to a family's capacity to handle challenges that could affect its structure and function. A higher PS score indicates lower problem-solving ability, which may increase parenting stress. Our findings underscore the relevance of parental problem-solving abilities in reducing parenting stress. Without professional support to enhance parental DTT skills, they may encounter greater difficulties in addressing child-rearing challenges. Therefore, professional institutions should provide psychological support and technical guidance to families in order to implement effective family-based interventions. Strategies such as structured play and sport activities can facilitate the healthy development of children with ASD and contribute to the establishment of a positive parenting cycle.

Finally, our study revealed the independent factors influencing parenting stress, such as family dysfunction (specifically, problem-solving, affective responsiveness, and affective involvement abilities), child-related factors (including gender, language disorder, and ADHD), caregiver-related factors (including gender, education, and DTT skills), intervention method, and duration. As protective factors: (1) the intervention strategy based on King's goal attainment theory and improved parental DTT skills reaffirms the effectiveness of our hospital-family collaborative intervention; and (2) among family functioning factors, problem-solving ability helps families manage challenges during intervention, and affective responsiveness and involvement can enhance empathy and mutual support among family members (29), reducing accumulated conflict. As risk factors: (1) male children often show more externalizing behaviors like aggression and hyperactivity (30); (2) language disorder or ADHD can worsen communication and behavior challenges (31); (3) male caregivers may feel more stress due to social roles and emotional regulation differences; and (4) caregivers with less education often face limited access to resources and difficulties in implementing interventions. These factors may contribute to increased parenting stress. Therefore, intervention plans should address both children's developmental needs and family psychological support. It is important to use hospital-family collaboration instead of a single hospital intervention, extend the intervention duration to ensure the optimized effects; and family-based intervention should enhance parental emotional interaction and problem-solving skills to support both children's improvement and family adaptation.

## Conclusions

This study is the first to apply a DTT intervention based on King's goal attainment theory within a hospital-family collaboration framework. It shows promising results in improving core symptoms in children with ASD and family functioning, thus lowering parenting stress. The DTT teaching emphasized to primary caregivers during the in-hospital intervention phase, served as a critical foundation for effective ongoing family-based training. Simultaneously, Specialized nurses played a key role by expanding caregivers' ASD knowledge, enhancing their DTT skills, offering psychological support, and evaluating children's outcomes.

However, several limitations exist. First, as a small-sample, single-center study, the findings require further validation through larger sample sizes, multicenter investigations, and diverse racial and ethnic populations to minimize potential biases. Second, a three-month short-term family intervention yielded only immediate improvements, whereas sustained long-term effects necessitate extended follow-up periods. Third, certain individual and contextual factors related to parenting styles are not examined in this study, which may introduce potential bias.

Despite these limitations, the findings highlight the value of ongoing family involvement in DTT and provide useful insights for enhancing health management for children with ASD. Future long-term follow-up validation is recommended to better assess the effectiveness and applicability of this hospital-family collaboration DTT intervention.

## Data Availability

The raw data supporting the conclusions of this article will be made available by the authors, without undue reservation.
